# Balancing harms and benefits of service mammography screening programs: a cohort study

**DOI:** 10.1186/bcr3090

**Published:** 2012-01-09

**Authors:** Donella Puliti, Guido Miccinesi, Marco Zappa, Gianfranco Manneschi, Emanuele Crocetti, Eugenio Paci

**Affiliations:** 1 Clinical and Descriptive Epidemiology Unit, ISPO - Cancer Prevention and Research Institute, via San Salvi 12, 50135 Florence, Italy

## Abstract

**Introduction:**

The use of screening mammography is still under debate within the medical community. The aim of this study is to define a balance sheet of benefits (breast cancer mortality reduction) and harms (overdiagnosis) for mammography screening programs.

**Methods:**

We compared breast cancer incidence and mortality in two cohorts of women, defined as 'attenders' or 'non-attenders' on the basis of the individual attitudes towards screening, who were invited to the first round of the Florentine screening program. The effects of screening exposure on breast cancer incidence and mortality were evaluated by fitting Poisson regression models adjusted for age at entry, marital status and deprivation index. We performed a sensitivity analysis excluding 34 women not responding to the invitation with a breast cancer diagnosis in the following six months.

**Results:**

In total, we included 51,096 women aged 50 to 69 years invited at the first screening round (1991 to 1993) and followed-up for breast cancer incidence and mortality until 31 December 2007 and 31 December 2008, respectively The estimate of mortality reduction varies from 45% among 50 to 59 year-old women up to 51% among 60 to 69 year-old women. The estimate of overdiagnosis, according to the cumulative-incidence method, is an additional 10% of all breast cancer cases among 60 to 69 year-old women screened.

**Conclusions:**

Comparing the breast cancer mortality and breast cancer incidence between attenders and non-attenders, we have determined that the overall cost to save one life corresponds to no more than one over-diagnosed tumor (from 0.6 to 1 depending on the selection criteria of the cohort), even if a residual self-selection bias cannot be excluded.

## Introduction

The efficacy of mammography screening programs has been assessed in large randomized trials conducted in the 1970s and 1980s both in Europe and in North America [1-3]. Moreover, the effectiveness of population-based screening in the context of routine health care has been proven in many European countries using different approaches (incidence-based mortality and case-control approaches) [4-8].

Nevertheless, the use of screening mammography is still under debate and the effectiveness of mammography screening programs in reducing breast cancer mortality was recently questioned on the basis of two observational studies [9,10]. In addition, some authors have highlighted potential negative side effects of mammography screening. In particular they have raised the problem of overdiagnosis [11,12], considered to be the most important harm associated with early detection. Although criticisms of mammography screening were based on dubious methodology - that has been widely criticized [13,14] - the estimation of the absolute benefits and harms of mammography screening for breast cancer remains a subject of discussion [15-17]. Therefore an evaluation based on the experience of service screening programs in Europe is urgent.

The best method to assess the impact of service screening is the cohort approach, a study design including subjects followed up in time both for their experience of screening and for the outcomes. In this paper breast cancer incidence and mortality in the group of women invited to the Florentine screening program are presented. Two cohorts of women, identified on the basis of the individual attitude towards screening, are compared in order to obtain an estimate of the ratio between benefits and harms of mammography screening, in terms of absolute numbers of lives saved and absolute numbers of tumors overdiagnosed.

## Materials and methods

The Florentine screening program began in 1991, offering high-quality mammography every 2 years to all resident women aged 50 to 69 years [18]. The attendance rate was 56% at the prevalence round and it has increased to about 70% in recent years. Performance indicators including diagnosis and treatment are collected annually under a national survey carried out by the National Center for Screening Monitoring [19] on behalf of the Italian Ministry of Health.

The cohort included the 52,282 women 50 to 69 years old who were invited to the first screening round of the Florentine screening program (1991 to 1993). The fact of having had a previous diagnosis of breast cancer is a reason for non-response to invitation. Therefore we excluded 264 women with a prior breast cancer diagnosis as registered in the Tuscany cancer registry [20]. Moreover, we excluded 922 women who had migrated outside the screening area before the second invitation as registered in the municipality register.

The screening histories of all women in the cohort, including the dates of invitations and the dates of all their screening tests following the invitations, if any, were extracted from the local computerized screening database. Screening exposure was defined on the basis of attendance at the first two rounds and the women were classified as:

1) *frequent attenders*, if they responded to both invitations,

2) *irregular attenders*, if they responded to only one invitation,

3) *never attenders*, if they not responded to any of the first two invitations.

For the women invited only to the first round (*n *= 5,757) since they were not eligible for the second round (due to cancer diagnosis, age limit, recent spontaneous screening mammography, and so on) screening exposure was defined as frequent or never attenders on the basis of the attendance at the first round.

In order to verify the correlation between attendance at the first two rounds and at successive rounds, the whole screening history of the women up to 31 December 2008 was collected. A total of 84% of women who were classified as 'never' did not attend any test in the study period. For the women classified as 'frequent' and 'irregular' the average attendance at the successive rounds, calculated as the total number of performed tests compared to the total number of invitations received from the third round onwards, is 90% and 67%, respectively. Because of the similar screening attendance frequent and irregular attenders were combined together in the 'attenders' category (that is, all women who responded to at least one invitation in the first two rounds), in order to obtain more reliable estimates for the balance sheet.

All women were followed-up for breast cancer incidence until 31 December 2007 through a linkage with the Tuscan cancer registry [20] and pathology reports. All women were followed-up for vital status and cause of death until 31 December 2008 through a linkage with the regional mortality registry and with the list of residents [21]. Person-years at risk were counted from the date of first invitation to the date of event (breast cancer diagnosis/breast cancer death) or to the date of censoring (death due to other causes, emigration from Tuscany or end of follow-up). Standardized incidence and mortality rates were calculated using the European standard population.

In order to verify the comparability of attenders and non-attenders, information was collected on marital status (at December 1995) and deprivation index (national census 1991 data) for all women in the cohort. The deprivation index was constructed at the national level to measure relative socio-economic disadvantage [22] and it is available at the level of census section (about 70 women for each section). Three socio-economic classes were defined using tertiles of the index distribution in the Florentine area.

Furthermore, in order to enhance the comparability of these two groups, we performed a sensitivity analysis excluding 34 women not responding to the invitation who had a breast cancer diagnosis in the following six months. The underlying assumption is that these women were already undergoing diagnostic assessment when they received the invitation to screening.

The local ethics committee has authorized this study as an observational retrospective study, aimed at public health outcome research; therefore no consent by subjects was needed according to the national law.

### Statistical Analysis

In order to evaluate benefits and harms of mammography screening programs, breast cancer mortality reduction and overdiagnosis was taken into consideration. The effects of screening exposure on breast cancer mortality and breast cancer incidence were evaluated by fitting Poisson regression models adjusted for age at entry (five-years class), marital status and deprivation index.

Due to the length of our incidence follow-up (15.4 years median value), the breast cancer incidence rate ratio can be considered a valid estimate of overdiagnosis only for women 60 to 69 years old at entry, according to the cumulative-incidence method [23]. In the cumulative-incidence method a comparison is made of the cumulative incidence among a group of women who were screened with the cumulative incidence in a non-screened group over the same time period. The comparison should be carried out several years (at least five) after screening ends, in order to take into account the lead time attributable to screening [23]. Indeed, in a previous paper [24] we showed that 90% of incremental cases are expected to be decremental five years after screening stops. The younger cohort, 50 to 59 years old at the entry, will be 65 to 74 years old at the end of the study period. Therefore they were still being invited to screening or the follow-up period after the last screening was not long enough. The older cohort, 60 to 69 years old at the entry, will be 75 to 84 years old at the end of the study period, that is, they were followed for 5 to 14 years after the end of screening. Therefore, the follow-up period was long enough to take into account the lead time and to provide a correct estimate of overdiagnosis.

The balance sheet was constructed by estimating the number of lives saved and the number of overdiagnosed cases among 1,000 60 to 69 year-old women screened until the screening age limit (69 years) and followed for 15 years. The absolute number of lives saved was calculated in a two-step process:

1) the risk of breast cancer death in the absence of screening was estimated from the breast cancer mortality rates observed in the cohort of non-attenders.

2) the number of lives saved was calculated by applying our mortality reduction estimate to the number of breast cancer deaths expected in the absence of screening.

The absolute number of overdiagnosed cases was calculated using a similar procedure.

## Results

In total, we studied 51,096 women who were 50 to 69 years old at the first invitation of the Florentine screening program. Overall 64% of the women (*n *= 32,544) responded to at least one invitation in the first two rounds. This is the group of 'attenders' composed of 48% frequent attenders and 16% irregular attenders. The 'non-attenders' did not responded to any of the first two invitations and constituted 36% of the total (*n *= 18,552). Over the study period, attenders aged 50 to 59 and 60 to 69 years at the first invitation had 6.0 and 3.3 screening mammograms on average, respectively.

As shown in Table 1 attenders were significantly younger than non-attenders (mean age was 59.1 and 60.9 years, respectively, *P *< 0.0001). The distribution of marital status was very different by screening exposure: 70% of attenders were married compared to only 57% of non-attenders (*P *< 0.0001). Furthermore, attenders and non-attenders differed by socio-economic status: the percentage of women who lived in a deprived area (that is, a census section classified in the third tertile of the deprivation index distribution) was 31.7% and 36.0% for attenders and non-attenders, respectively (*P *< 0.0001). Overall, 1,583 (4.9%) breast cancers were diagnosed during the follow-up period among the attenders and 782 (4.2%) among non-attenders. The number of breast cancer deaths observed in the cohort was 184 (0.6%) and 218 (1.2%) for attenders and non-attenders, respectively.

**Table 1:** Women characteristics by screening exposure

	Attenders	Non-attenders	
N° of women	32,544	18,552	
			
Mean age at entry	59.1	60.9	p < 0.0001
			
Marital status (%)			
Married	70.0%	57.3%	
Unmarried	8.0%	15.0%	
Divorced	2.3%	3.2%	
Widow	19.7%	24.5%	p < 0.0001
			
Deprivation index (%)			
First tertile	34.0%	31.3%	
Second tertile	34.3%	32.7%	
Third tertile	31.7%	36.0%	p < 0.0001
			
N° (%) of breast cancer	1,583 (4.9%)	782 (4.2%)	
In situ	143 (0.4%)	36 (0.2%)	
Invasive	1,440 (4.4%)	746 (4.0%)	
			
N° (%) of BC deaths	184 (0.6%)	218 (1.2%)	

**Figure 1:**
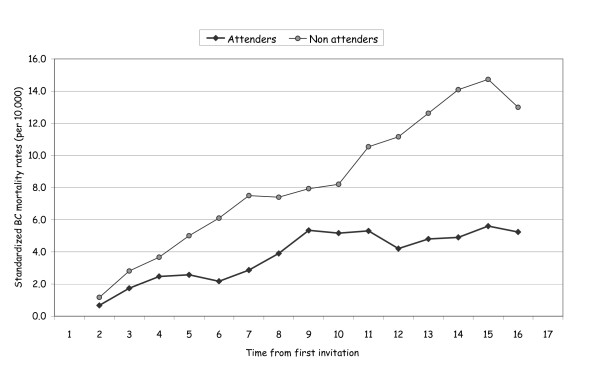
**Standardized mortality rates from breast cancer (per 10,000) by time from first invitation (three-years moving averages)**. Women 50 to 69 years old at entry.

All women were followed up for vital status with a median follow-up time of 16.5 years. In total, we observed 9,641 deaths from all causes of which 402 were deaths from breast cancer. Standardized mortality rates from breast cancers were 3.6 and 7.5 per 10,000 for screened and never attenders, respectively. In Figure 1 we show the standardized mortality rates from breast cancer (per 10,000) by time from first invitation. Mortality rates of attenders and non-attenders were similar in the first three to four years after invitation and diverged progressively over time.

All women were followed up for incidence with a median follow-up of 15.4 years. In total, we observed 2,365 incidence breast cancers (2,186 invasive and 179 *in situ*). Standardized breast cancer incidence rates (per 1,000) were 3.4 and 3.0 for attenders and non-attenders, respectively.

**Figure 2:**
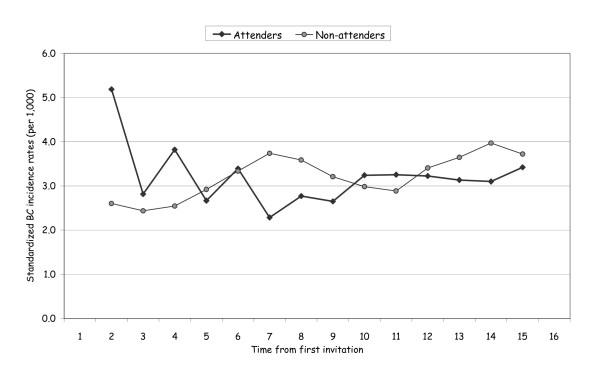
**Standardized breast cancer incidence rates (per 1,000) by time from first invitation (three-year moving averages)**. Women 60 to 69 years old at entry.

In Figure 2 we report the standardized breast cancer incidence rates (per 1,000) by time from first invitation for women 60 to 69 years old at entry. During the first years we observed the incidence peak in the attenders due to the prevalence round. The differences between the two groups were gradually reabsorbed as the women were no longer actively invited.

In Table 2 the number of cases, person-years and rate for breast cancer mortality and incidence are shown by age at entry and exposure to screening. The rate ratio was adjusted for age, marital status and deprivation index. The estimate of mortality reduction varies from 45% among 50 to 59 year olds up to 51% among 60 to 69 year olds. The estimate of overdiagnosis, according to the cumulative-incidence method, is an additional 10% of breast cancer cases (invasive and *in situ*) among those screened. The estimate of overdiagnosis for invasive breast cancer only was 5% (1.05; 95%CI: 0.93 to 1.18).

**Table 2:** Number of cases, person-years, rates and rate ratios for breast cancer mortality and incidence by age at entry among attenders and non-attenders

		Breast cancer mortality			
**Age at entry**	**Exposure**	**BC deaths**	**Person years**	**BC mortality rate** **(per 10,000)**	Adjusted rate ratio (*)

50-59	Non-attenders	77	113 409	6.8	1
	Attenders	90	270 399	3.3	0.55 (0.41 - 0.75)

60-69	Non-attenders	141	151 615	9.3	1
	Attenders	94	233 543	4.0	0.49 (0.38 - 0.64)

		**Breast cancer incidence**			

**Age at entry**	**Exposure**	BC cases (**)	**Person years**	**BC incidence rate (per 1,000)**	Adjusted rate ratio (*)

50-59	Non-attenders	321	105 635	3.0	1
	Attenders	838	249 896	3.4	1.15 (1.01 - 1.31)

60-69	Non-attenders	461	142 547	3.2	1
	Attenders	745	216 309	3.4	1.10 (0.98 - 1.23)

(*) Adjusted for age, marital staus and deprivation index. (**) Include *in situ *and invasive BC cases

Using the rates for non-attenders, the 15-year risk of breast cancer (*in situ *and invasive) for women 60 to 69 years old was 5.7% and the risk of dying from breast cancer in the same age class was 1.8%. Therefore, screening 1,000 women 60 to 69 years old until the screening age limit (performing an average of three screening mammograms each) may prevent about 9 breast cancer deaths out of 18 expected and could lead to an overdiagnosis of about 6 cases out of 57 expected, including carcinoma *in situ*, leading to a balance of 0.6 tumors overdiagnosed for every life saved.

### Sensitivity analysis

We performed a sensitivity analysis excluding 34 women not responding to the invitation with a breast cancer diagnosis in the following 6 months. The estimate of mortality reduction remains essentially unchanged (43% and 48% for the 50 to 59 and 60 to 69 year olds) but the estimate of overdiagnosis increases to 15% for all breast cancers (10% for invasive only), so leading to a balance of one tumor overdiagnosed for every life saved.

## Discussion

Although mammography screening is one of the screening programs most carefully studied, it continues to be one of the most debated issues within the medical community [25]. The effectiveness of mammography screening programs in reducing breast cancer mortality was recently questioned [9,10]. Moreover, the problem of overdiagnosis with mammography screening has been raised by several authors who have tried to quantify overdiagnosis of breast cancer [23]. The estimates obtained vary widely, depending on the method used, leading to a much heated debate between supporters of opposite positions [15,16].

The main challenge in quantifying the reduction in mortality and the overdiagnosis from screening programs is to provide valid comparison groups. In service screening, in fact, a control group is not available since the entire target population is invited to be screened. In most studies the method used to overcome this problem is to estimate the incidence or mortality expected in the absence of screening, modelling the incidence or mortality rates observed before the start of the screening program [24,26-29]. However, the estimate of expected rates is subject to a strong statistical uncertainty and it strongly influences the estimate of the final outcome (mortality reduction or overdiagnosis).

In the present study, a comparison was made between two observed cohorts, the cohort of women who responded to at least one invitation in the first two rounds (attenders) and the cohort of women who did not respond to any invitation in the first two rounds (non-attenders), thus overcoming the problem of estimating the expected. On the other hand, due to the well-known self-selection bias (women who accept the invitation to screening may have a baseline risk for breast cancer incidence and mortality different from that of women who do not accept the invitation to screening) the comparison between attenders and non-attenders can be biased. A correction for self-selection bias was made using the information about marital status and socio-economic status of the women [30]. Furthermore, in order to enhance the comparability of these two groups, we performed a sensitivity analysis excluding 34 women who had not responded to the invitation and who had a breast cancer diagnosis in the following 6 months (the underlying assumption is that these women were already undergoing diagnostic assessment when they received the invitation to screening). Nevertheless we cannot exclude the presence of a residual self-selection bias.

It should be noted that the issue of comparability between attenders and non-attenders is the main limitation of this study and it can influence either mortality or incidence results. As far as mortality is concerned, several studies suggested that non-attenders have a higher baseline risk of breast cancer mortality compared to a control group [31] even if other authors found the opposite [32]. As far as we know, there is no consistent evidence in the literature about different underlying incidence risks in attenders compared with non-attenders. In our dataset, attenders belong to a higher socio-economic class and are more frequently married than never attenders. The first factor is probably associated with higher underlying incidence while the second one with lower underlying incidence, resulting in an unknown net effect. Therefore, it is not clear in which direction the self-selection bias could have influenced our results.

Screening exposure was defined on the basis of attendance at the first two rounds. A tendency to maintain the same behavior over time (called 'attitude towards screening') was noted in the Italian annual screening survey [33] and also in other countries [34]. The above data support this assumption, too. Indeed 84% of women who did not respond to the first two rounds still did not respond to the successive rounds and 91% of women who responded to at least one invitation in the first two rounds also responded to at least one more invitation in successive rounds. Nevertheless, a misclassification of exposure may occur in a small number of women. The misclassification of exposure between attenders and non-attenders could lead to a bias towards the null hypothesis, which means a possible underestimation of both mortality reduction and overdiagnosis.

The aim of this paper is to define a balance sheet of benefits (breast cancer mortality reduction) and harms (overdiagnosis) for service mammography screening. It has been estimated that, if 1,000 women 60 to 69 years old at entry were screened until the age limit (performing an average of three screening mammograms each) about 9 women will benefit from the screening, because they will avoid dying from breast cancer, while about 6 women will be overdiagnosed and treated needlessly (the expected figures in the absence of screening are 18 breast cancer deaths and 57 *in situ *and invasive breast cancer cases). Therefore, the human cost to save one life corresponds to 0.6 overdiagnosed tumours.

Our final balance sheet (between 0.6 and 1 tumor overdiagnosed for every life saved) is consistent with the results reported by Duffy on the basis of both experimental and observational data from the Swedish Two-County trial and from the Breast Screening Program in England [16]. However, estimates from other authors, Welch [24] and Gotzsche [12], give a much higher number of overdiagnosed tumors. It could be said that the first step to comparing estimates from different studies and to formulating a common balance sheet is to share a common methodology. The estimate of overdiagnosis is strongly dependent on the method used for the lead time adjustment. The cohort approach with a long follow-up after screening ends, as in this paper, is the best approach to take into account the bias due to lead time without using any statistical adjustment. Moreover, as far as we know, this is the first observational cohort study of invited women comparing both breast cancer incidence and mortality between those who participated and those who did not participate in screening (in other words assessing both overdiagnosis and mortality reduction from the same cohort of women).

The balance sheet should be based on reliable estimates both of mortality and on overdiagnosis. This implies that agreement is reached on the best method to obtain these estimates, on updating these estimates continuously and in reaching context-specific estimates.

Our estimate of overdiagnosis resulted in an additional 10% of *in situ *and invasive breast cancer cases and it was reduced to 5% when invasive cancers only were considered. These data show that a large part of overdiagnosis is due to carcinoma *in situ *even if we cannot draw conclusions about whether and how the detection of carcinoma *in situ *could lead to an incidence reduction in invasive cases. Therefore, the actual estimate of overdiagnosis to be used in the balance sheet should include carcinoma *in situ*.

Furthermore, in order to reduce the burden of overdiagnosis, it is essential to understand better the natural history of small lesions. Indeed, it should be recalled that the information available in order to predict tumor progression and aggressiveness is very poor, especially for carcinoma *in situ*. Further research is needed to improve understanding of the markers of tumor progression and so to reduce the burden of treatment in early breast cancer cases which is the most important and needed consequence of screening.

## Conclusions

The best method to assess the impact of service screening is the cohort approach, a study design including subjects followed up in time both for their experience of screening and for the outcomes. As far as we known, this is the first observational cohort study of invited women comparing both breast cancer incidence and mortality between those who participated and those who did not participate in screening. Comparing breast cancer mortality and breast cancer incidence between attenders and non-attenders, we have estimated that the overall cost to save one life corresponds to no more than one overdiagnosed tumor (from 0.6 to 1 depending on the selection criteria of the cohort), even if a residual self-selection bias cannot be excluded.

## Abbreviations

Ne: not estimable.

## Competing interests

The authors declare that they have no competing interests.

## Authors' contributions

PD and ManG performed the data collection and quality control; PD performed the statistical analysis; PD, MicG, ZM and PE conceived the study design, interpreted the results and wrote the paper; CE was involved in revising the manuscript critically for important intellectual content. All authors read and approved the final manuscript.
